# Bartonella clarridgeiae *and* B. henselae *in Dogs, Gabon*

**DOI:** 10.3201/eid1012.040359

**Published:** 2004-12

**Authors:** Vijay A.K.B. Gundi, Olivier Bourry, Bernard Davoust, Didier Raoult, Bernard La Scola

**Affiliations:** * Faculté de Médecine, Marseille, France;; † Centre International de Recherches Médicales, Franceville, Gabon;; ‡ Direction Régionale du Service de Santé des Armées, Lyon, France

**Keywords:** letter, Bartonella, cat scratch disease, bartonellosis, dog, Gabon

**To the Editor:** The genus *Bartonella* contains several recently described species, many of which are emerging human pathogens. Human infections are mostly due to *Bartonella henselae* and *B. quintana*. Like many vectorborne disease agents, *Bartonella* species have a natural cycle. This cycle contains a reservoir host, in which *Bartonella* species cause an intraerythrocytic bacteremia, and a vector, which transmits the bacteria from the reservoir host to a new susceptible host (usually the uninfected reservoir host) (*1*). In the case of *B. quintana* and *B. bacilliformis*, the natural host is human. In *Bartonella* diseases, humans act as accidental hosts. Among the nonhuman *Bartonella* species that infect humans, *B. henselae* is most commonly encountered and usually causes cat-scratch disease. However, several cases of infections in humans attributable to other *Bartonella* species, including *B. elizabethae*, *B. grahamii*, *B. vinsonii arupensis*, *B. vinsonii berkhoffii*, and possibly *B. clarridgeiae*, have been reported (*1*). Isolation of *Bartonella* species in animals that have contact with humans can help identify new human pathogens or new diseases. We report results of isolation of *Bartonella* spp. from the blood of 258 dogs in Gabon.

The study was performed in the Ogooué-Ivindo province of Gabon, a country of Central Africa with an equatorial climate. Blood samples were taken from dogs in the town of Mékambo and in all villages connecting Mékambo and Mazingo (nine villages) and Mékambo and Ekata (seven villages) during July and August 2003. Each dog brought by its owner for the study was weighed and sedated by injection with 50 μg/kg of medetomidine (Pfizer Santé Animal, Orsay, France). After the dog was examined, a blood sample was drawn from the jugular vein by Vacutainer (Becton Dickinson, Meylan, France). Each dog was tattooed with an identification number and given both antihelminthic and external antiparasitic treatments. During the examination, the dogs were treated with care; upon completion of the examination, the dogs were given 250 μg/kg of the reversal agent atipamezola (Pfizer Santé Animal) intramuscularly. A physical examination form and a questionnaire were completed for each test participant by its owner. A total of 258 dogs (155 males and 103 females) were examined and had blood samples drawn during the study. All animals were of mixed breeds and were 6 months to 14 years old (average 3 years 1 month). The Vacutainer tubes were kept on ice until blood samples were dispensed into cryotubes and frozen in liquid nitrogen. Samples were stored at –80°C until isolation attempts were made on Columbia agar (Biomérieux, Marcy l'étoile, France) as described previously (*2*). In this study, six *Bartonella* isolates were obtained and identified as *B. clarridgeiae* (five isolates) and *B. henselae* (one isolate), by internal transcribed spacer amplification and sequencing (*3*).

*B. vinsonii* subsp. *berkhoffii* was the first *Bartonella* species found in dogs (*1*). Isolation of *B. clarridgeiae* (*4**,**5*) and *B. washoensis* (*6*) in dogs was recently reported. Infection of dogs by other *Bartonella* species was also detected in the DNA of *B. henselae* (*7**,**8*), *B. clarridgeiae* (*7*), and *B. elizabethae* (*8*). The presence of these *Bartonella* species is not surprising, since *Ctenocephalides felis*, the vector of *B. henselae* in cats, has a wide range of hosts, including the domestic dog. However, attempts to isolate this species in samples collected from 211 dogs in the United Kingdom failed (*9*). *Bartonella* species are supposedly difficult to isolate in dogs because of a low concentration of bacteria in the blood (*1*). This supposition was apparent in our study; we identified approximately 100 bacterial colonies per milliliter of blood from three of the six dogs in our study. From the other three dogs in our study, including the dog infected with *B. henselae*, we identified two to four bacterial colonies per milliliter of blood.

Most of the data pertaining to *Bartonella* have been obtained in the United States and Europe. Increasingly, *Bartonella* infections are being reported in Africa, especially in southern Africa (*10*). We report here the first isolation of *B. henselae* from a dog and the first isolation of *B. clarridgeiae* in Central Africa. That dogs also act as reservoirs of *B. henselae* likely has implications in Africa where HIV infections are prevalent.
